# A2 reactive astrocyte‐derived exosomes alleviate cerebral ischemia–reperfusion injury by delivering miR‐628

**DOI:** 10.1111/jcmm.70004

**Published:** 2024-08-19

**Authors:** Yingju Wang, He Li, Hanwen Sun, Chen Xu, Hongxue Sun, Wan Wei, Jihe Song, Feihong Jia, Di Zhong, Guozhong Li

**Affiliations:** ^1^ Department of Neurology The First Affiliated Hospital of Harbin Medical University Harbin Heilongjiang People's Republic of China; ^2^ Department of Neurology Heilongjiang Provincial Hospital Harbin Heilongjiang People's Republic of China; ^3^ Department of Emergency Rui Jin Hospital Affiliated to Shanghai Jiao Tong University School of Medicine Shanghai People's Republic of China

**Keywords:** A2 astrocyte, blood–brain barrier, cerebral ischemia, IRF5, microglial polarization, miR‐628, NLRP3, pyroptosis, S1PR3

## Abstract

Ischemia and hypoxia activate astrocytes into reactive types A1 and A2, which play roles in damage and protection, respectively. However, the function and mechanism of A1 and A2 astrocyte exosomes are unknown. After astrocyte exosomes were injected into the lateral ventricle, infarct volume, damage to the blood–brain barrier (BBB), apoptosis and the expression of microglia‐related proteins were measured. The dual luciferase reporter assay was used to detect the target genes of miR‐628, and overexpressing A2‐Exos overexpressed and knocked down miR‐628 were constructed. qRT–PCR, western blotting and immunofluorescence staining were subsequently performed. A2‐Exos obviously reduced the infarct volume, damage to the BBB and apoptosis and promoted M2 microglial polarization. RT–PCR showed that miR‐628 was highly expressed in A2‐Exos. Dual luciferase reporter assays revealed that NLRP3, S1PR3 and IRF5 are target genes of miR‐628. After miR‐628 was overexpressed or knocked down, the protective effects of A2‐Exos increased or decreased, respectively. A2‐Exos reduced pyroptosis and BBB damage and promoted M2 microglial polarization through the inhibition of NLRP3, S1PR3 and IRF5 via the delivery of miR‐628. This study explored the mechanism of action of A2‐Exos and provided new therapeutic targets and concepts for treating cerebral ischemia.

## 1 INTRODUCTION

Ischemic stroke (IS) is the second leading cause of death worldwide. It is also the leading cause of long‐term adult disability.^1^ IS is usually caused by occlusion of the middle cerebral artery, which supplies blood to the brain.^2^, ^3^ In recent years, intravenous thrombolysis and arterial thrombectomy techniques have revolutionized the treatment of ischemic stroke.^4^ Although blood flow is recovered through medical intervention, reperfusion or restored blood flow causes an inflammatory response, oxidative stress (OS), excitatory amino acids and cell death. This is called cerebral ischemia–reperfusion (I/R) injury.^5^, ^6^, ^7^ Cerebral I/R injury can increase cerebral infarction volume, damage the blood–brain barrier (BBB) and aggravate cerebral edema.^8^ Therefore, reducing cerebral I/R injury in the brain is a key step in IS treatment.

Pyroptosis is an inflammatory form of regulated cell death that occurs after cerebral I/R injury. The canonical pathway of pyroptosis is mediated by caspase‐1. Activated caspase‐1 releases the N‐terminal domain of gasdermin D (GSDMD‐N), which destroys the cell membrane and results in the release of large amounts of IL‐1β and IL‐18.^9^ The inflammatory corpuscles NOD‐like receptor thermal protein domain associated protein 3 (NLRP3) and apoptosis‐associated speck‐like protein containing CARD (ASC) stimulate pyroptosis by recruiting and activating caspase‐1.^10^ Inflammatory factors released during pyroptosis cause neuroinflammation and cell death.^11^ Moreover, damage to the BBB after cerebral I/R injury further exacerbates brain edema and exacerbates neurological impairment and mortality. Sphingosine‐1‐phosphate (S1P) receptor 3 (S1PR3) is a downstream G protein that regulates BBB damage after IS. Thus, inhibiting S1PR3, which is an important target of IS treatment, can protect the BBB.^12^, ^13^ Furthermore, microglia, the immune hallmark of neuroinflammation, differentiate into different subtypes (M1 and M2) following I/R injury. M1‐type neurotoxic microglia and M2‐type neuroprotective microglia release proinflammatory and anti‐inflammatory factors such as TNF‐α and IL‐10, respectively.^14^, ^15^ Interferon regulatory factor 5 (IRF5) plays a crucial role in microglial polarization. Inhibiting IRF5 can promote the polarization of M1 microglia to the M2 phenotype and alleviate cerebral I/R injury.^16^ In conclusion, NLRP3, S1PR3 and IRF5 can serve as important therapeutic targets in cerebral I/R injury.

Astrocytes are key factors for promoting various brain functions, including metabolic transformation, synaptogenesis, scar production and BBB formation in the central nervous system. Many of these functions can be partially achieved via the secretion of exosomes that act as signalling conveyors in cellular communication.^17^, ^18^, ^19^ Following cerebral ischemia, reactive astrocytes differentiate into A1 and A2 subtypes and provide toxic or protective substances to neurons, respectively. A1 reactive astrocytes can upregulate many classical complement cascade components and induce neuronal death, while A2 reactive astrocytes produce neurotrophic factors and promote neuronal growth.^20^, ^21^, ^22^, ^23^ However, the functions of exosomes secreted from these A1‐ and A2‐reactive astrocytes have not yet been elucidated.

In the present study, we found that miR‐628 significantly reduced cerebral I/R injury. The underlying mechanism may involve alleviating pyroptosis, protecting the BBB, and intervening in the polarization of microglia. Moreover, A2‐Exos were also found to protect against cerebral I/R injury through similar mechanisms (including reducing infarct volume and brain oedema content, alleviating cell apoptosis and promoting M2 microglial polarization). A significant increase in the expression of miR‐628 was noted in A2‐Exos compared with normal astrocyte‐derived exosomes and A1‐Exos. Bioinformatics analysis (TargetScan 8.0, https://www.targetscan.org/vert_80/) predicted NLRP3, S1PR3 and IRF5 as the target genes of miR‐628. Additionally, we used overexpression and knockdown techniques to construct A2‐Exos with different miR‐628 concentrations to explore the mechanism underlying the protective effects of A2‐Exos.

## 2 METHODS

### 2.1 Cell lines

Rat primary microglia, rat primary astrocytes and human embryonic kidney (HEK) 293 T cells were purchased from Procell Co., Ltd. The cells were grown in Dulbecco's modified Eagle's medium (DMEM, Gibco) supplemented with 10% fetal bovine serum. All cells were cultured at 37°C in a 5% CO_2_ incubator.

A1‐ and A2‐reactive astrocytes were obtained as previously described.^24^ Briefly, supernatants obtained from microglia stimulated with 40 ng/mL lipopolysaccharide (Sigma) or 40 ng/mL interleukin‐4 (Biotime) for 3 days were used to induce A1‐ and A2‐reactive astrocytes. The induction of A1‐ and A2‐reactive astrocytes was completed after 3 days.

### 2.2 Cell transfection

A vector containing a miR‐628 mimic (artificially synthesized miR‐628)/miR‐628 inhibitor (artificially synthesized miRNA that inhibits miR‐628)/miR‐628 negative control mimic (ineffective miR‐628 mimic)/miR‐628 negative control inhibitor (ineffective miR‐628 inhibitor) was transfected into cells with Lipofectamine™ 3000 (Thermo Fisher Scientific) according to the manufacturer's instructions. After 6 h, transfection was terminated, and the culture medium was replaced.

### 2.3 Astrocyte‐derived exosome isolation and characterization

Exosome‐free complete medium (Umibio) was added to the primary astrocyte culture at 90% confluence. After 24 h, the supernatant was collected for the isolation of exosomes. Astrocyte‐derived exosome isolation was performed at 4°C using an exosome isolation kit (Umibio) as described in our previous study.^25^ The isolated exosomes were resuspended in PBS at a 1000 μg/mL concentration. For the morphology investigation, transmission electron microscopy (TEM) was used. The particle size of the exosomes was measured by nanoparticle tracking analysis (NTA). Exosome‐specific markers, such as ALG‐2 interacting protein X (ALIX), tumour susceptibility gene 101 protein (TSG101), and CD9, were detected via western blotting.

### 2.4 Rat transient middle cerebral artery occlusion (tMCAO) reperfusion model and neurological function score

All experimental procedures were conducted following the existing rules of the First Affiliated Hospital of Harbin Medical University (Approval number: 2022127) and the National Institutes of Health Guidelines for the Care and Use of Laboratory Animals. SPF‐grade SD rats (weight, 260 ± 20 g) were obtained from Liaoning Changsheng Biotechnology Co., Ltd. All animals were housed in a controlled environment (temperature, 25°C, under a 12‐h light/dark cycle) and were provided standard nutrition and water.

The rat model of tMCAO was induced as previously described.^25^ The suture was not inserted in the sham group animals. The rats were allowed to fast for 12 h before the tMCAO operation. Finally, the rats were euthanized by intraperitoneal injection of sodium pentobarbital at 1, 3, and 7 days after reperfusion. The ischemic cortical area of the brain tissue was collected for subsequent experiments. According to previous reports, miR‐628 mimic and miR‐628 negative control (10 μg) or exosomes containing the same mimic were injected by intracerebroventricular injection before tMCAO. We ensured that each part of the experiment was completed using the same batch of animals. To minimize the number of experimental animals, total RNA and protein were collected from the brain tissue of the same rat. During the study, 9 rats of 154 in total, died because of subarachnoid haemorrhage or cerebral I/R injury before euthanasia (Figure S1). The neurological scores of the rats were recorded as described above to evaluate neurological function before sacrifice.^12^

### 2.5 2,3,5‐Triphenyte‐trazolium chloride (TTC) staining

The brain tissue was fixed on a pre‐cooled mould and sliced continuously starting from the frontal pole. Each brain tissue sample was cut into six sections of 2 mm thickness and stained with 2% TTC (Solarbio).

### 2.6 Measurement of the brain water content (BWC)

Brain tissue sections stained with TTC were weighed to record the wet weight, dried for 24 h at 90 °C and then weighed again to record the dry weight. The BWC was calculated as a percentage using the following equation: BWC (%) = (wet weight−dry weight)/wet weight × 100.

### 2.7 Evans blue assay

A 2% solution of Evans blue in normal saline (4 mL/kg of body weight) was injected intraperitoneally. The stain was allowed to circulate for 6 h. After euthanasia, brain tissue was collected and homogenized on ice, after which 50% trichloroacetic acid was added. Next, the samples were centrifuged for 20 min at 3000 rpm. Evans blue staining was measured with a spectrophotometer at 620 nm and quantified according to a standard curve.

### 2.8 Dual‐luciferase reporter assay

Fragments of the 3′ untranslated regions (3′UTRs) of NLRP3, S1PR3 and IRF5 containing wild‐type (WT) or mutant miR‐628 binding sites were amplified and inserted into the pmirGLO vector (Promega). The insertion was verified. HEK293T cells were transfected, and 24 h after transfection, the activities of firefly and Renilla luciferase were measured using a multifunctional microplate reader.

### 2.9 RNA extraction and real‐time polymerase chain reaction (RT‐PCR)

Total RNA was extracted with TRIzol (Thermo Fisher Scientific, USA). Specific reverse transcription and q‐PCR of miRNAs were initiated using the FastKing first‐strand synthesis kit (Tiangen) and SYBR Green kit (Tiangen). GAPDH and U6 were used as endogenous references for mRNA and miRNA, respectively. The primers for miR‐628 and U6 were purchased from Tiangen Biotechnology. The other sequences of the primers used were as follows: NLRP3, forward, 5′‐ CTCGCATTGGTTCTGAGCTC‐3′ and reverse, 5′‐AGTAAGGCCGGAATTCACCA‐3′; S1PR3, forward, 5′‐ TGTCTCCAACAGTGTGGT‐3′ and reverse, 5′‐CAGCACATCCCAATCAGAAG‐3′; IRF5, forward, 5′‐GGAGTAGGGAGGATGTTTATT‐3′ and reverse, 5′‐AACTACTACCAAACCACCRCT‐3′; and GAPDH, forward, 5′‐ACTCCCATTCTTCCACCTTTG‐3′ and reverse, 5′‐CCCTGTTGCTGTAGCCATATT‐3′.

### 2.10 Western blotting

Brain tissue lysates were prepared by dissociating them in RIPA buffer (Boster). Total protein was isolated, and the protein concentration was measured using the BCA method. Of the protein sample, 30 μg was subjected to SDS–PAGE. Immunoblotting was performed using a polyvinylidene fluoride (PVDF) membrane. After the transfer, the membrane was incubated in blocking solution (10% milk) at room temperature for 1 h, followed by washing in TBST and incubation in appropriately diluted primary antibody solution overnight at 4°C. All the antibodies used were commercial against ALIX (ab186429, Abcam, 1:1000), TSG101 (ab125011, Abcam, 1:1000), CD9 (ab307085, Abcam, 1:1000), C3 (21337‐1‐AP, Proteintech, 1:10,000), S100A10 (11250‐1‐AP, Proteintech, 1:1000), C‐Caspase‐3 (66470‐2‐lg, Proteintech, 1:2000), Bcl2 (68103‐1‐Ig, Proteintech, 1:2000), BAX (60267‐1‐lg, Proteintech. 1:10000), iNOS (80517‐1‐RR, Proteintech, 1:2000), ARG1 (16001‐1‐AP, Proteintech, 1:5000), NLRP3 (ab263899, Abcam, 1:1000), S1PR3 (DF4869, Affinity, 1:1000), IRF5 (10547‐1‐AP, Proteintech, 1:2000), ASC (sc‐514414, Santa Cruz, 1:500), C‐Caspase‐1 (sc‐56036, Santa Cruz, 1:500), GSDMD‐N (DF13758, Affinity, 1:1000), IL‐1β (AF4006, Affinity, 1:1000), IL‐18 (10663–1‐AP, Proteintech, 1:10,000), MMP9 (ab228402, Abcam, 1:2000), occludin (ab216327, Abcam, 1:2000), TNF‐α (60291‐1‐Ig, Proteintech, 1:1000), IL‐10 (60269‐1‐Ig, Proteintech, 1:1000), CD16 (16559‐1‐AP, Proteintech, 1:1000), CD206 (ab64693, Abcam, 1:1000) and β‐actin (TA‐09, ZSGB, 1:2000). The next day, the samples were washed in TBST, probed with specific secondary antibodies and detected using an enhanced chemiluminescence (ECL) detection system (Biosharp, China). Densitometric analysis of the bands was performed using ImageJ software (National Institutes of Health).

### 2.11 Immunofluorescence (IF) assay

Brain tissue was perfused with PBS, followed by 4% paraformaldehyde before harvesting. Fixed tissues were embedded in an optimal cutting temperature compound, and frozen sections were prepared at a thickness of 7 μm. The sections were blocked in blocking buffer (Beyotime, P0260, China) and incubated with primary antibodies overnight at 4°C and with TRITC‐ or FITC‐conjugated secondary antibodies for 2 h at room temperature. The following primary antibodies were used: C3 (21337‐1‐AP, Proteintech, 1:100), S100A10 (21337‐1‐AP, Proteintech, 1:100), occludin (ab216327, Abcam, 1:50), ZO1 (21773‐1‐AP, Proteintech, 1:1000), GSDMD‐N (DF13758, Affinity, 1:100), iNOS (80517‐1‐RR, Proteintech, 1:100), ARG1 (16001‐1‐AP, Proteintech, 1:100), CD16 (16559‐1‐AP, Proteintech, 1:100), CD206 (ab64693, Abcam, 1:100), caspase‐3 (66470‐2‐lg, Proteintech, 1:100) IBA1 (sc‐32725, Santa Cruz, 1:50 and GTX638147, Gene Tex, 1:200) and NeuN (ab177487, Abcam, 1:300). Slides were counterstained with DAPI (ab104139, Abcam) for 10 min. Images were acquired using a fluorescence microscope.

### 2.12 Terminal deoxynucleotidyl transferase dUTP nick end labeling (TUNEL) staining

Following immunofluorescence, TUNEL solution (TUNEL assay kit, Roche, Switzerland) was added to the frozen sections, which were then incubated in the dark at 37°C for 1 h. Images were acquired using a fluorescence microscope.

### 2.13 Statistical analysis

All the data are expressed as the means ± standard deviations and were analysed with GraphPad 8.3.0 software. One‐way ANOVA followed by Tukey's post hoc test was applied to compare the differences among groups; *p* < 0.05 suggested that the difference was significant.

## 3 RESULTS

### 3.1 miR‐628 alleviates cerebral I/R injury

**FIGURE 1:**
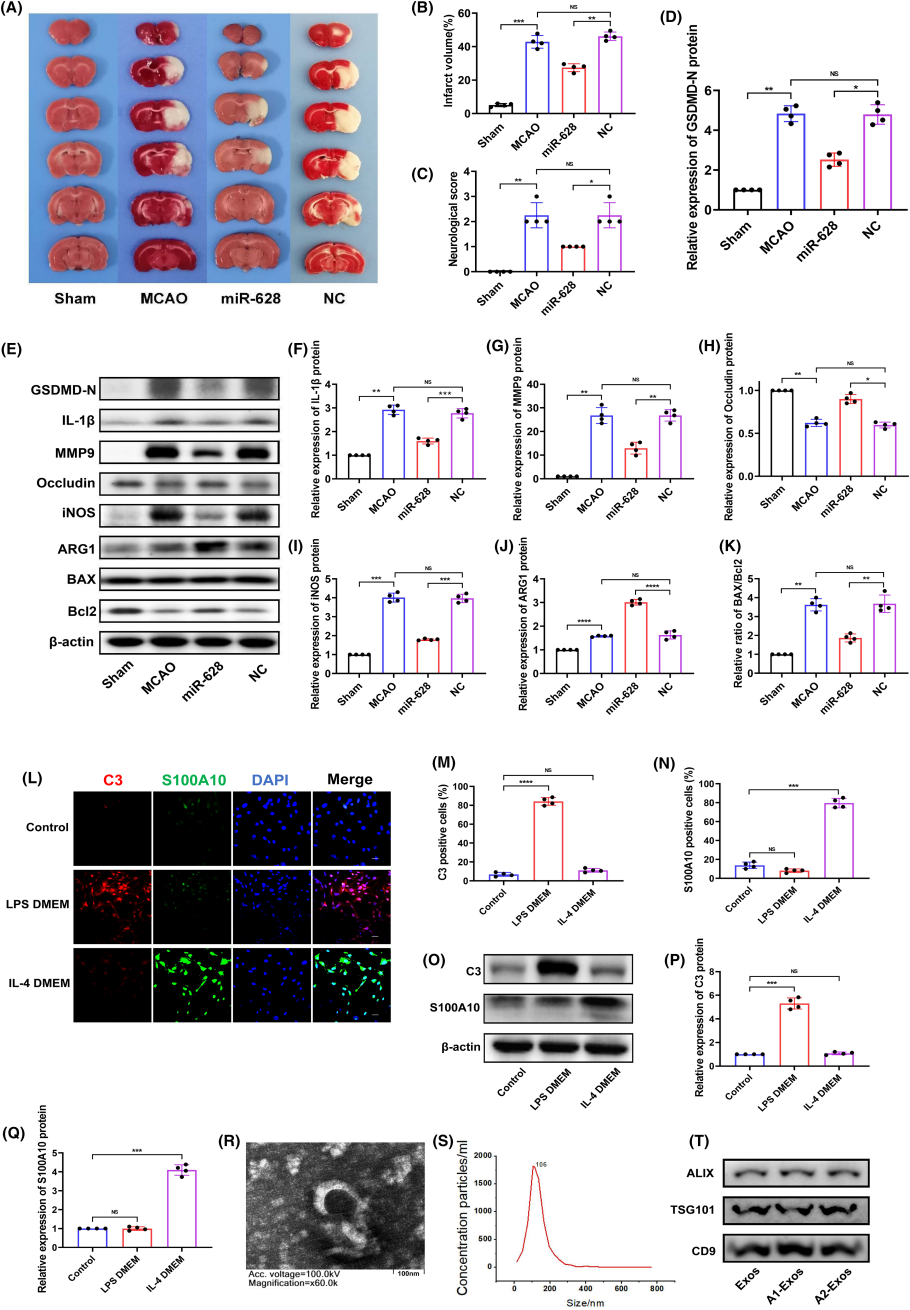
The treatment effect of miR‐628 on cerebral I/R injury and reactive astrocyte exosome identification. (A, B) The infarct volume was determined by TTC staining (*n* = 4). (C) The statistical analysis of neurological score (*n* = 4). (D–K) Protein expression of GSDMD‐N, IL‐1β, MMP9, occludin, iNOS, ARG1, BAX and Bcl2 after miR‐628 treatment was measured by western blot (*n* = 4). (L–N) IF staining was conducted to analyse C3 and S100A10 after phenotypic induction for 3 days. (O–Q) Protein expression of C3 and S100A10 after phenotypic induction for 3 days. (R) Typical TEM images of astrocyte‐derived exosomes. Scale bar = 100 nm. (S) Content and size of exosomes shown by NTA. (T) The levels of the astrocyte exosome markers ALIX, TSG101 and CD9 were detected by western blotting. The results are presented as the means ± SDs. *p < 0.05; **p < 0.01; ***p < 0.001; ****p < 0.0001; NS, not significant following one‐way ANOVA plus Tukey's post hoc test.

To investigate the role of miR‐628 in cerebral I/R injury, a miR‐628 mimic or miR‐628 negative control (NC) was injected into the lateral ventricle of rats after inducing injury. To identify the neurorestorative effect of miR‐628, several parameters, including infarct volume, pyroptosis, damage to the BBB, microglial polarization and apoptosis‐related proteins, were analysed. The structure of the BBB is composed of the tight junction proteins occludin and ZO1. They form tight junctions between vascular endothelial cells across the central nervous system. After cerebral I/R injury, MMP9 is released, and it degrades tight junction proteins, including occludin and ZO1, causing BBB leakage and poor prognosis.^26^ Microglial polarization in the different groups was detected using specific protein markers expressed by microglia. iNOS and ARG1 are protein markers of the M1 and M2 microglial subtypes, respectively.^27^ Infarcts were observed in all the experimental groups except the sham group, which confirmed the successful establishment of the tMCAO model (Figure 1A). Compared to those in the sham group, the infarct volume, neurological score and protein levels in the NC group did not significantly change (Figure 1A–K). Treatment with miR‐628 significantly reduced the infarct volume, neurological score and levels of pyroptosis‐, BBB‐, microglial polarization‐ and apoptosis‐related proteins (Figure 1A–K). These results suggest that miR‐628 has a protective effect on cerebral I/R injury, and the underlying mechanism may be related to pyroptosis, the BBB and microglial polarization.

### 3.2 Identification of astrocyte type and exosomes

A1‐ and A2‐reactive astrocytes were identified based on the expression of C3 and S100A10, which are specific markers of A1‐ and A2‐reactive astrocytes, respectively.^20^ C3 expression increased when astrocytes were cultured with LPS, and the expression of S100A10 increased upon IL‐4 induction (Figure 1L–Q). The morphology of astrocyte‐derived exosomes was detected by TEM (Figure 1R), and the particle size of the exosomes was detected by NTA (Figure 1S). Western blotting was used to analyse exosomes secreted by astrocytes in the medium, and ALIX, TSG101 and CD9 were detected (Figure 1T).

### 3.3 A2‐Exos alleviate cerebral I/R injury and cell apoptosis

**FIGURE 2:**
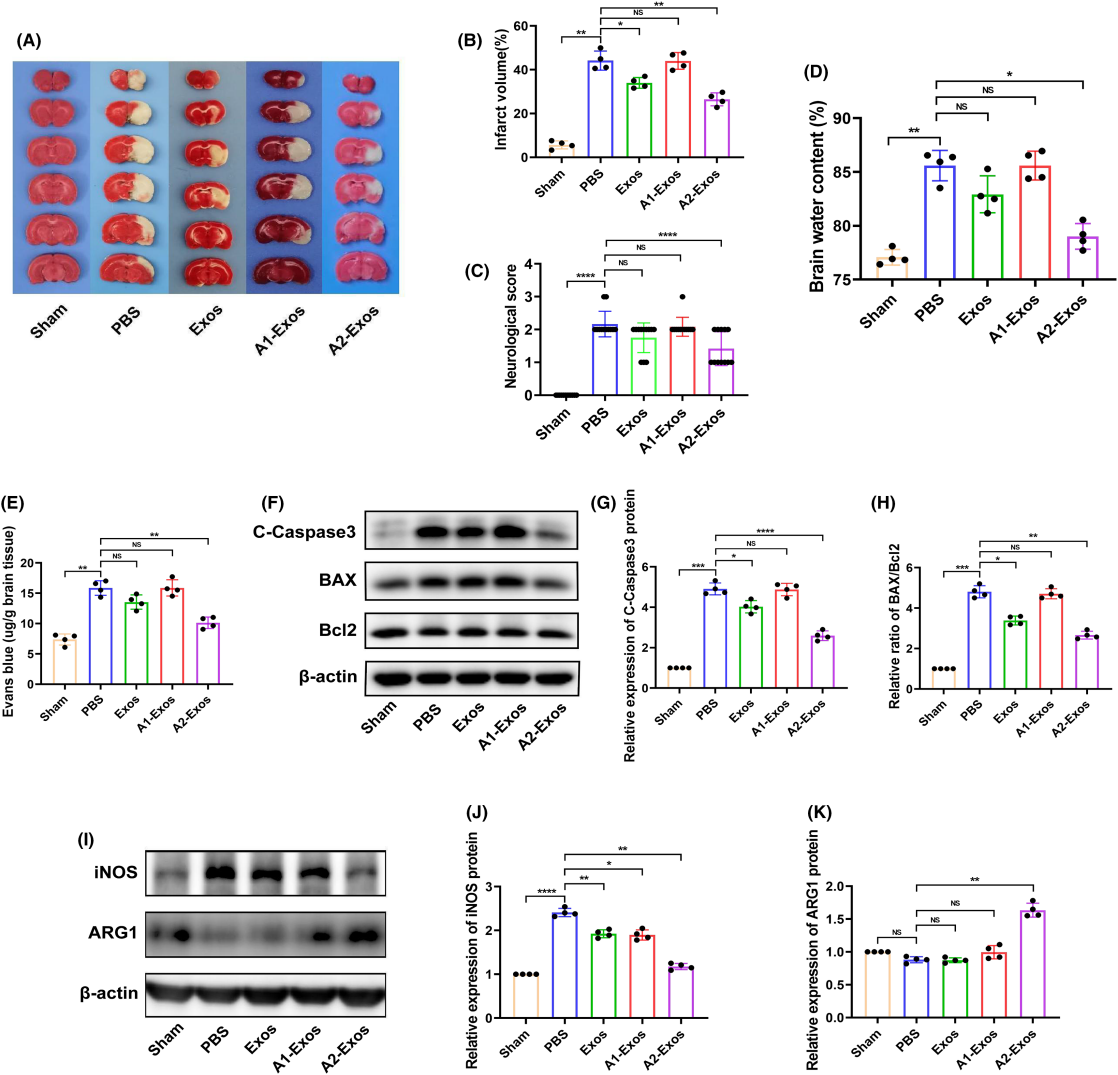
Treatment effects of astrocyte exosomes on cerebral I/R injury and microglial polarization. (A, B) The infarct volume was determined by TTC staining (*n* = 4). (C) The statistical analysis of neurological score (*n* = 12). (D) Measurement of brain water content (*n* = 4). (E) Evans blue staining (*n* = 4). (F–H) The protein expression of C‐Caspase‐3, BAX and Bcl2 after astrocyte exosome treatment was measured by western blot (*n* = 4). (I–K) Expression levels of iNOS and ARG1 protein after astrocyte exosome treatment were measured by western blotting (*n* = 4). The results are presented as the means ± SDs. *p < 0.05; **p < 0.01; ***p < 0.001; ****p < 0.0001; NS, not significant following one‐way ANOVA plus Tukey's post hoc test.

To explore the effect of astrocyte‐derived exosomes, 10 μL (10 μg) of exosomes secreted from different types of astrocytes or the same volume of PBS were separately injected intracerebroventricularly into the brains of tMCAO rats. Compared to control exosomes (with PBS treatment), normal astrocyte‐derived exosomes alleviated infarct volume and brain oedema and reduced Evans blue staining. The apoptosis‐related protein C‐Caspase‐3 and the ratio of Bax/Bcl‐2 were also decreased (Figure 2A–H). A2‐Exos had similar effects and significantly decreased the infarct volume, degree of brain oedema, amount of Evans blue dye, the protein level of C‐Caspase‐3 and ratio of Bax/Bcl‐2. In addition, the A2‐Exo group exhibited improved neurological function (Figure 2A–H). These results indicate that A2‐Exos have a neuroprotective effect on cerebral I/R injury.

### 3.4 A2‐Exos promote M2 microglial polarization

Compared to those in the PBS‐treated group, the protein levels of iNOS were significantly lower in the groups treated with normally derived astrocyte exosomes, A1‐Exos and A2‐Exos (Figure 2I,J). Additionally, treatment with A2‐Exos increased the protein level of ARG1 (Figure 2I,K). The results showed that A2‐Exos can significantly promote the polarization of microglia towards the M2 phenotype. Interestingly, treatment with both miR‐628 and A2‐Exos had similar protective effects on cerebral I/R injury. Next, we explored whether the mechanism underlying this neuroprotective effect of A2‐Exos is achieved through the delivery of miR‐628.

### 3.5 NLRP3, S1PR3 and IRF5 are target genes of miR‐628 and are highly expressed in A2‐Exos

**FIGURE 3:**
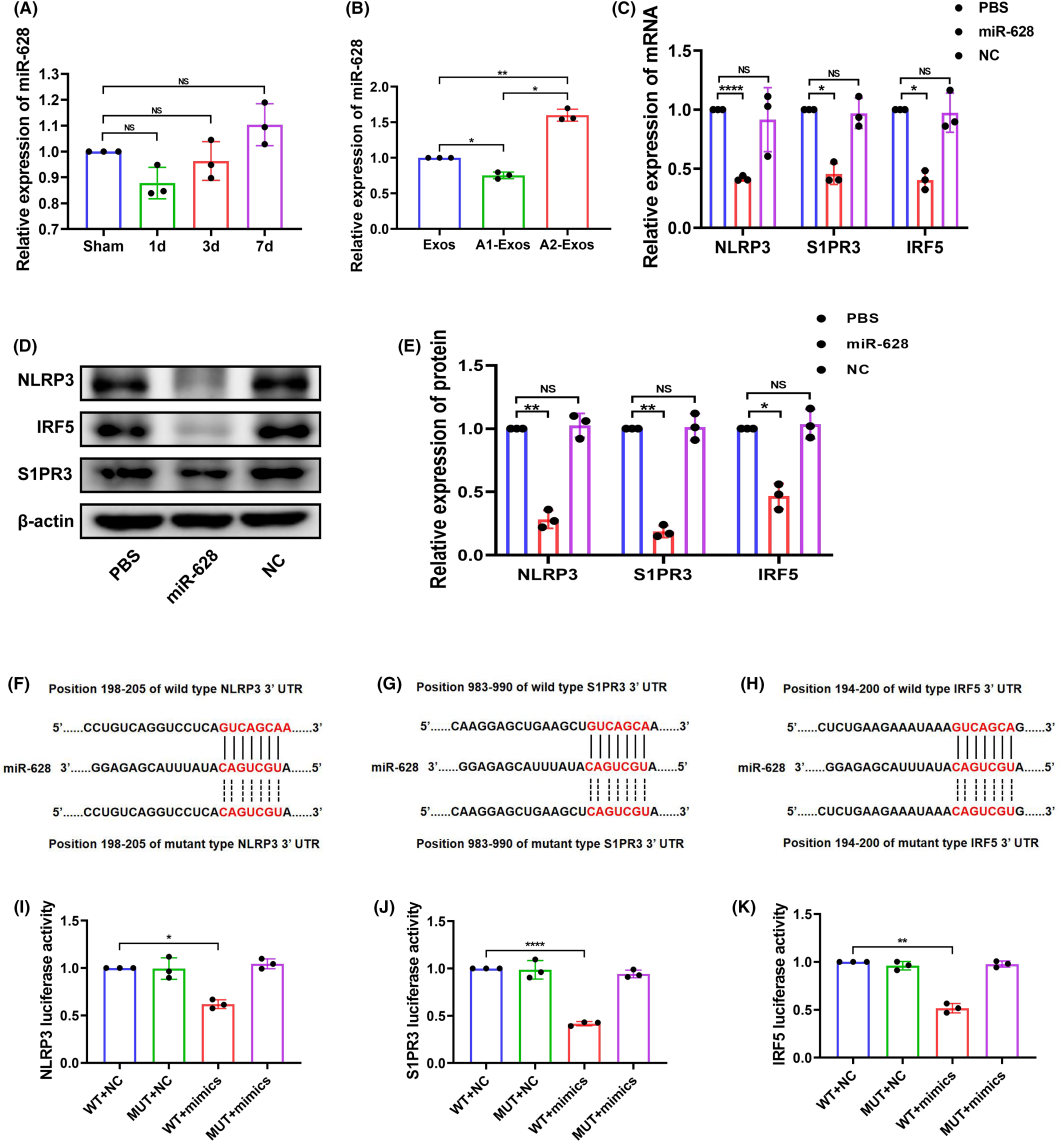
The expression level of miR‐628 and the target genes of miR‐628. (A) Expression level of miR‐628 varies with time after cerebral I/R (*n* = 3). (B) Expression level of miR‐628 in exosomes of astrocytes with different response types (*n* = 3). (C) Expression levels of NLRP3, S1PR3 and IRF5 mRNA after intracerebroventricular injection of PBS, miR‐628 and NC (*n* = 3). (D, E) Expression levels of the NLRP3, S1PR3 and IRF5 proteins after intracerebroventricular injection of PBS, miR‐628 and NC (*n* = 3). (F–H) Binding sites of NLRP3, S1PR3, IRF5 and miR‐628. (I–K) Luciferase activity of HEK293T cells (*n* = 3). The results are presented as the means ± SDs. *p < 0.05; **p < 0.01; ***p < 0.001; ****p < 0.0001; NS. not significant following one‐way ANOVA plus Tukey's post hoc test.

After cerebral I/R injury, the expression level of miR‐628 in brain tissue changed slightly over time (Figure 3A). In A1‐Exos, the expression level of miR‐628 was lower than that in normal astrocyte‐derived exosomes. In contrast, the expression level of miR‐628 was significantly greater in the A2‐Exos (Figure 3B). NLRP3, S1PR3 and IRF5 were predicted to be target genes of miR‐628 by bioinformatics analysis via TargetScan 8.0. Therefore, intracerebroventricular injection of miR‐628 and miR‐628 NC was performed to explore the downstream regulatory effects on the abovementioned genes and proteins. The mRNA expression of NLRP3, S1PR3 and IRF5 was measured by qRT–PCR, and the results showed a decrease in the expression of these genes upon miR‐628 treatment following cerebral I/R injury (Figure 3C). We used western blotting to detect NLRP3, S1PR3 and IRF5 protein expression (Figure 3D,E). The results showed that, compared to the expression levels in the miR‐628 NC group, the miR‐628 group had significantly inhibited protein expression. The dual luciferase reporter assay results confirmed that miR‐628 upregulation decreased luciferase activity in HEK 293 T cells cotransfected with NLRP3, S1PR3 and the IRF5 WT 3′‐UTR (Figure 3F–K). These results indicated that miR‐628 negatively regulates NLRP3, S1PR3 and IRF5 expression by directly binding to their corresponding 3′‐UTRs.

### 3.6 miR‐628 content in exosomes is effectively regulated

**FIGURE 4:**
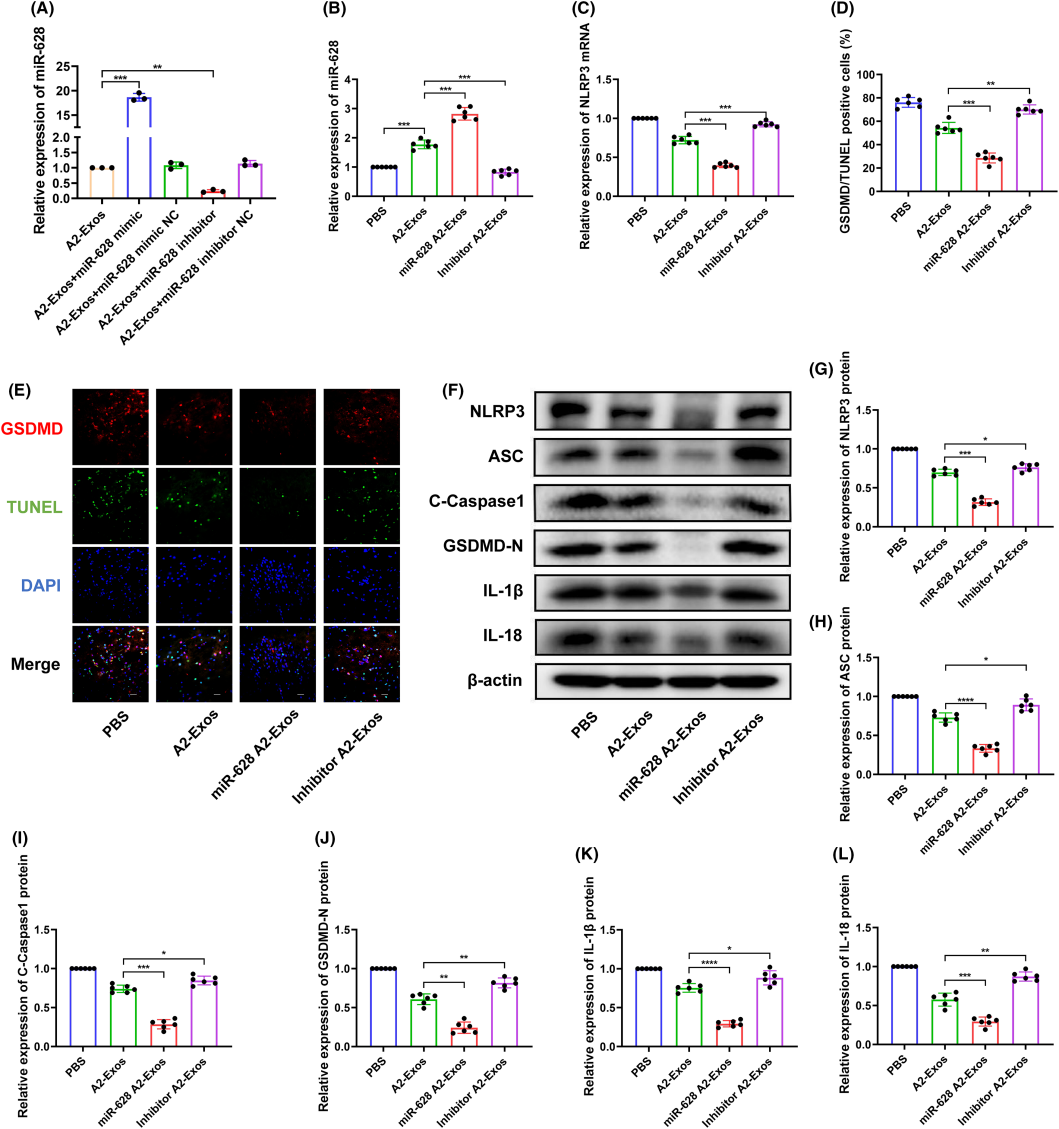
A2 reactive astrocyte exosomes alleviate pyroptosis by delivering miR‐628 after cerebral I/R injury. (A) Expression level of miR‐628 in exosomes of transfected A2 reactive astrocytes (*n* = 3). (B) Expression level of miR‐628 after different exosome treatments after cerebral I/R (*n* = 6). (C) Expression level of NLRP3 mRNA (*n* = 6). (D, E) IF staining was conducted to analyse representative images of GSDMD and TUNEL at 1 day after MCAO (×400 magnification, scale bar = 50 μm) (*n* = 6). (F–L) The protein levels of NLRP3, ASC, C‐Caspase‐1, GSDMD‐N, IL‐1β and IL‐18 were determined by western blot (*n* = 6). The results are presented as the means ± SDs. *p < 0.05; **p < 0.01; ***p < 0.001; ****p < 0.0001; NS, not significant following one‐way ANOVA plus Tukey's post hoc test.

qRT–PCR showed that exosomes from A2 reactive astrocytes transfected with the miR‐628 mimic (miR‐628 A2‐Exos) expressed higher levels of miR‐628 than those from control A2‐Exos. The exosomes from A2 reactive astrocytes transfected with the miR‐628 inhibitor (Inhibitor A2‐Exos) showed lower levels of miR‐628. Moreover, exosomes from A2 reactive astrocytes transfected with miR‐628 NC mimic and miR‐628 NC inhibitor did not show any change in the level of miR‐628 (Figure 4A). These A2‐Exos, miR‐628 A2‐Exos and inhibitor A2‐Exos were incorporated into subsequent experiments.

### 3.7 A2‐Exos alleviate pyroptosis through the delivery of miR‐628 after cerebral I/R injury

Compared to that in the PBS‐treated group, the level of miR‐628 expression in brain tissue was greater in the group treated with A2‐Exos, and compared to that in the A2‐Exos group, the level of miR‐628 expression was greater in the miR‐628 A2‐Exos group but lower in the inhibitor A2‐Exos group (Figure 4B). These results indicate that A2‐Exos can deliver miR‐628 to the affected tissue following cerebral I/R injury. Next, the levels of proteins associated with pyroptosis were analysed. A TUNEL assay was also performed to detect cell death. Compared to that in the A2‐Exo group, the level of NLRP3 mRNA was lower in the miR‐628 A2‐Exo group but greater in the inhibitor A2‐Exo group (Figure 4C). Immunofluorescence staining revealed that the percentage of GSDMD/TUNEL‐positive cells was lower in the miR‐628 A2‐Exos group but greater in the inhibitor A2‐Exos group (Figure 4D,E). Compared to those in the PBS‐treated group, the levels of NLRP3, ASC, C‐Caspase‐1, GSDMD‐N, IL‐1β and IL‐18 proteins were decreased in the miR‐628 A2‐Exos group and increased in the inhibitor A2‐Exos group (Figure 4F–L).

### 3.8 A2‐Exos decrease the degeneration of tight junction proteins through the delivery of miR‐628 after cerebral I/R injury

**FIGURE 5:**
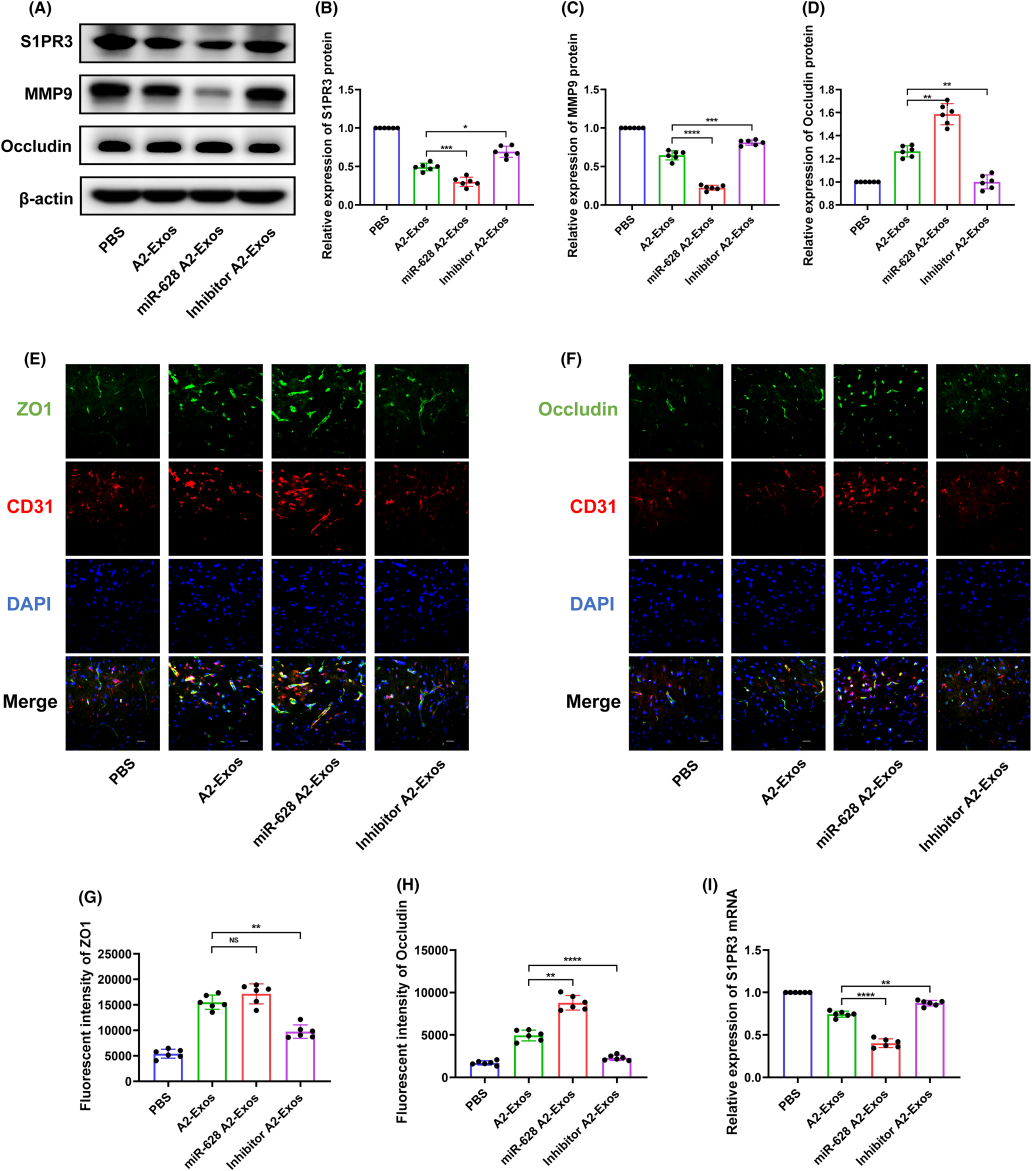
A2‐reactive astrocyte exosomes decrease the degeneration of tight junction proteins after cerebral I/R injury. (A–D) The protein levels of S1PR3, MMP9 and occludin were determined by western blot (*n* = 6). (E–H) IF staining of occludin and ZO1 at 1 day after MCAO. (×400 magnification, scale bar = 50 μm) (*n* = 6). (I) Expression level of SIPR3 mRNA (*n* = 6). The results are presented as the means ± SDs. *p < 0.05; **p < 0.01; ***p < 0.001; ****p < 0.0001; NS, not significant following one‐way ANOVA plus Tukey's post hoc test.

We used qRT–PCR to detect S1PR3 mRNA expression and western blotting to measure the protein levels of S1PR3, MMP9, and occludin. ZO1 and occludin were colocalized with CD31, a protein marker for endothelial cells. qRT–PCR showed that miR‐628 A2‐Exos treatment reduced S1PR3 mRNA levels more effectively than did A2‐Exos, and miR‐628 knockdown had the opposite effect (Figure 5I). Compared to those in the A2‐Exos group, the protein levels of S1PR3 and MMP9 were downregulated and the level of occludin was upregulated in the miR‐628 A2‐Exos group. In contrast, the protein levels of S1PR3 and MMP9 were increased, and that of occludin was decreased upon miR‐628 knockdown (Figure 5A–D). Fluorescent intensities of ZO1 and occludin were higher in the miR‐628 A2‐Exos group and decreased in the inhibitor A2‐Exos group (Figure 5E–H).

### 3.9 A2‐Exos promote M2 microglial polarization through the delivery of miR‐628 after cerebral I/R injury

**FIGURE 6:**
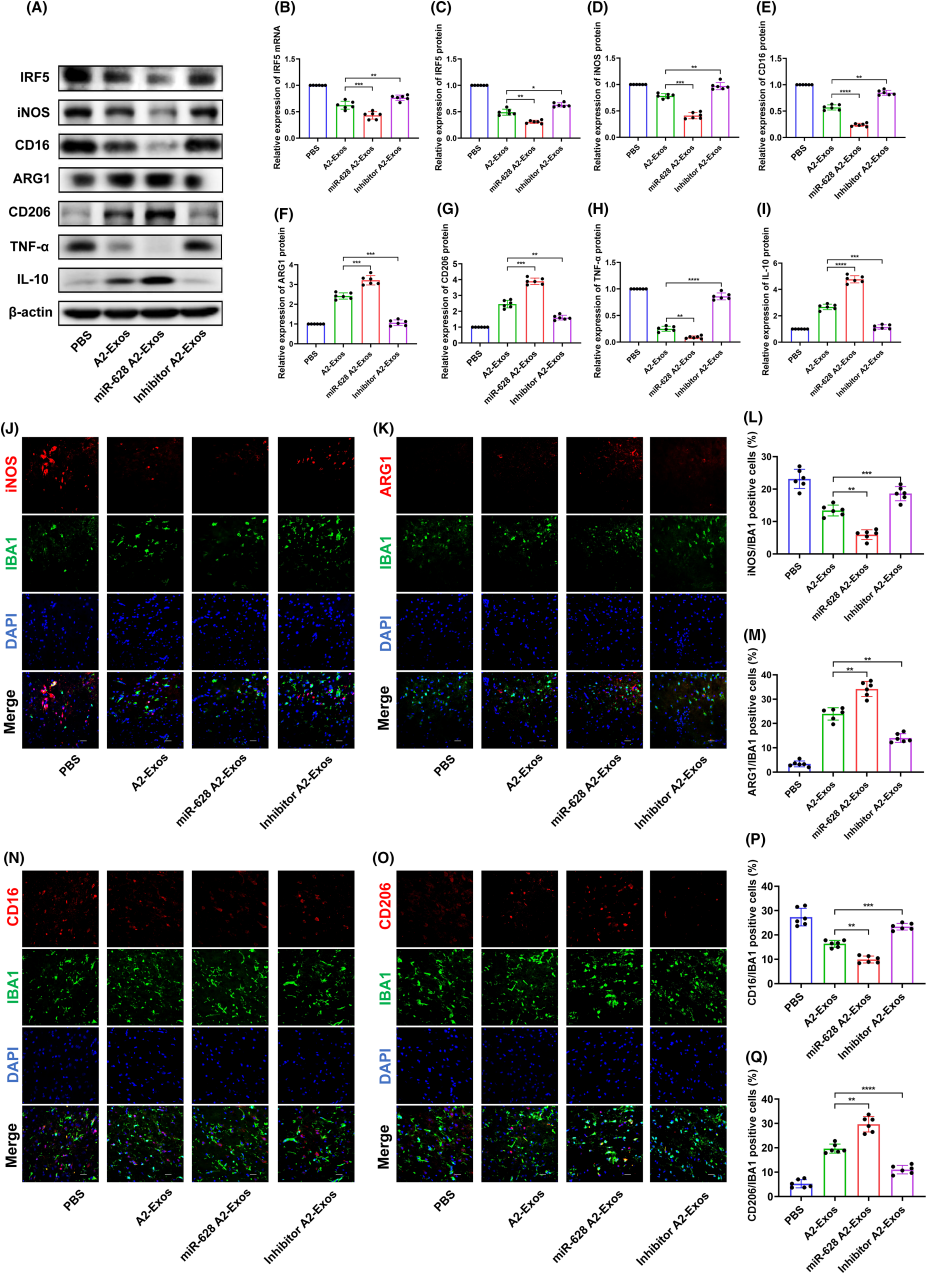
A2‐reactive astrocyte exosomes promote the polarization of M2 microglia after cerebral I/R injury. (A, C–I) The protein levels of IRF5, iNOS, CD16, ARG1, CD206, TNF‐α and IL‐10 were determined by western blotting (*n* = 6). (B) Expression level of IRF5 mRNA (*n* = 6). (J–M) IF staining of iNOS and ARG1, CD16 and CD206 at 1 day after MCAO. (IBA1, sc‐32,725, Santa Cruz, 1:50) (×400 magnification, scale bar = 50 μm) (*n* = 6). (N–Q) IF staining of CD16 and CD206 at 1 day after MCAO. (IBA1, GTX638147, Gene Tex, 1:200) (×400 magnification, scale bar = 50 μm) (*n* = 6). The results are presented as the means ± SDs. *p < 0.05; **p < 0.01; ***p < 0.001; ****p < 0.0001; NS, not significant following one‐way ANOVA plus Tukey's post hoc test.

qRT–PCR showed that miR‐628 A2‐Exos treatment reduced IRF5 mRNA levels more effectively than A2‐Exos, and treatment with the inhibitor A2‐Exos had the opposite effect (Figure 6B). To explore the regulatory effect of A2‐Exos on microglial polarization, we examined the M1 microglial markers iNOS and CD16 and the M2 microglial markers Arg1 and CD206. The levels of TNF‐α and IL‐10, secretory proteins specific to M1 and M2 microglia, respectively, were also tested.^27^ Compared to those in the A2‐Exo group, the protein expression levels of IRF5, iNOS, CD16 and TNF‐α were decreased, but the expression levels of ARG1, CD206 and IL‐10 were increased in the miR‐628 A2‐Exo group. Contrasting results were observed in the miR‐628 knockdown group (Figure 6A,C–I). We used double immunofluorescence staining to detect iNOS, CD16, ARG1 or CD206 and the microglial marker IBA1. IF staining showed that iNOS/IBA1‐positive cells and CD16/IBA1‐positive cells were decreased in number, while ARG1/IBA1‐positive cells and CD206/IBA1‐positive cells were increased in the miR‐628 A2‐Exos group. In the inhibitor A2‐Exos group, the numbers of iNOS/IBA1‐positive cells and CD16/IBA1‐positive cells were lower, and the numbers of ARG1/IBA1‐positive and CD206/IBA1‐positive cells were greater, suggesting that A2‐Exos promote M2 microglial polarization via miR‐628 delivery (Figure 6J–Q).

### 3.10 A2‐Exos protect against neuronal apoptosis through the delivery of miR‐628 after cerebral I/R injury

**FIGURE 7:**
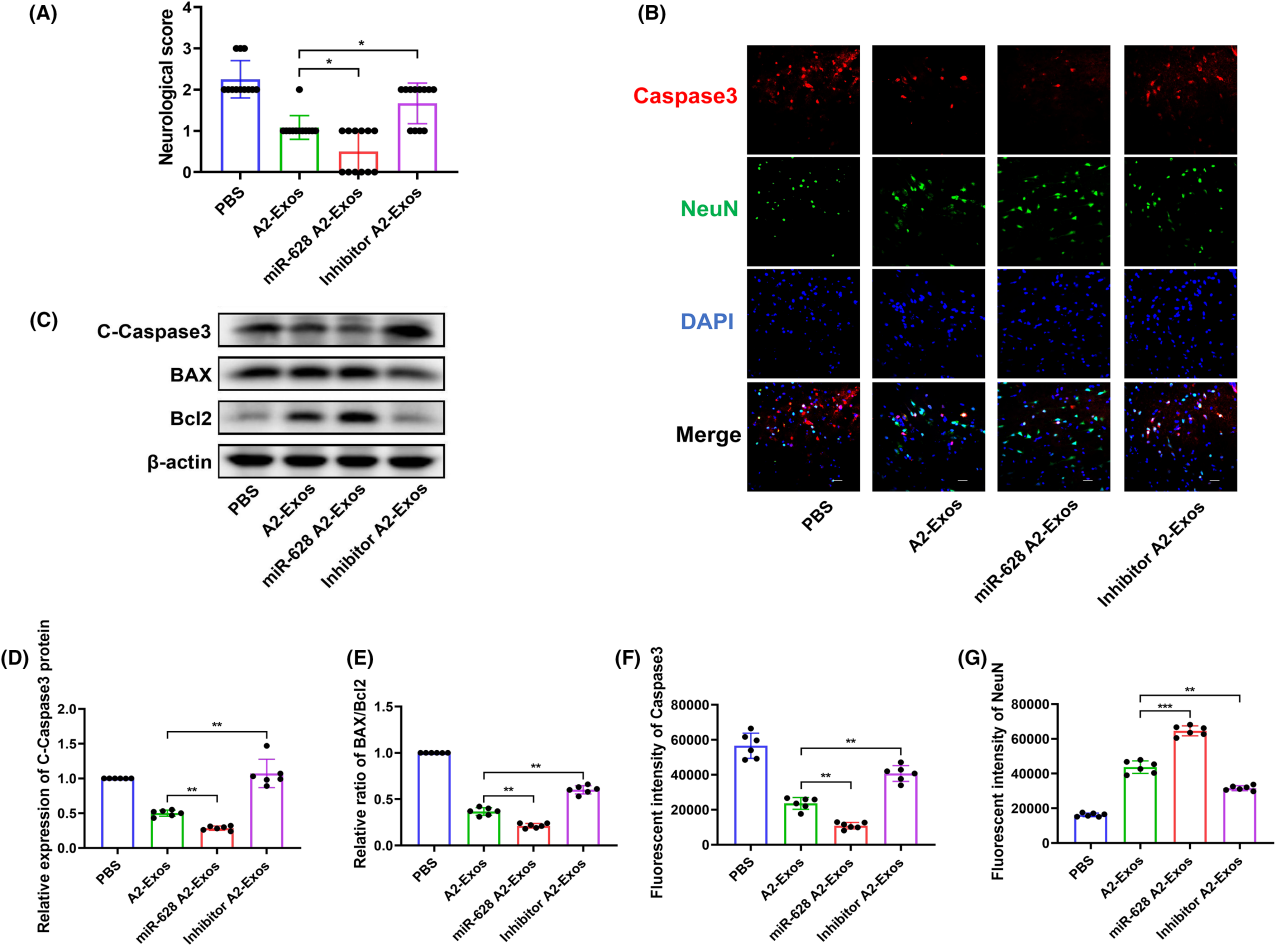
A2‐reactive astrocyte exosomes protect against cell apoptosis after cerebral I/R injury. (A) The statistical analysis of neurological score (*n* = 12). (C, D, E) The protein levels of C‐Caspase‐3, BAX and Bcl2 were determined by western blot (*n* = 6). (B, F, G) IF staining of Caspase‐3 and NeuN at 1 day after MCAO (×400 magnification, scale bar = 50 μm) (*n* = 6). Results are represented as means ± SDs. *p < 0.05; **p < 0.01; ***p < 0.001; ****p < 0.0001; NS. not significant following one‐way ANOVA plus Tukey's post hoc test.

To determine the neuroprotective effect of A2‐exos, the degree of neuronal apoptosis was measured. Compared to those in the A2‐Exos group, the protein level of caspase‐3 and the ratio of Bax/Bcl‐2 were decreased in the miR‐628 A2‐Exos group and increased in the inhibitor A2‐Exos group (Figure 7C–E). IF staining showed a decreased fluorescence intensity of caspase‐3 and a greater fluorescence intensity of NeuN in the miR‐628 A2‐Exos group. The opposite results were obtained upon miR‐628 knockdown (Figure 7B,F,G). Moreover, the neurological score showed the same trend (Figure 7A).

## 4 DISCUSSION

Astrocytes are key factors in the physiological and pathological processes underlying the formation of neurovascular units. These cells also produce nerve growth factor, neurotrophic factor and neurotrophins.^28^ The role of reactive astrocytes following cerebral ischemia is bidirectional. Previous studies have suggested that reactive A1 astrocytes secrete neurotoxins and release various complements that kill neurons. A2 reactive astrocytes were found to secrete protective factors that promote the remodelling of neurovascular units.^29^, ^30^ To date, the A1 and A2 phenotypes are not conclusive enough to classify reactive astrocytes. Additionally, there are only a few protein markers for determining the subtype of astrocytes, and to identify the astrocyte subtypes more accurately, other markers are needed.^31^ In the present study, we briefly grouped reactive astrocytes based on the available protein markers C3 and S100A10. Exosomes secreted from astrocytes can mediate intercellular communication. Previous studies have shown that astrocyte‐derived exosomes can alleviate neuronal damage by inhibiting autophagy.^17^ Moreover, astrocyte‐derived exosomes can reduce neuronal apoptosis.^32^ However, studies on the impact of exosomes produced from A1‐ and A2‐reactive astrocytes are rare. In this study, we compared the effects of exosomes obtained from different types of astrocytes after cerebral ischemia. Normal astrocyte‐derived exosomes showed protective effects against cerebral I/R injury. This observation is consistent with previous results. A1‐Exos did not have any significant protective or damaging effects. The reason could be the inability of the exosomes to completely substitute for cellular functions, which may require the involvement of microglia.^33^ Meanwhile, A2‐Exos significantly reduced the infarct volume, damage to the BBB, and pyroptosis and promoted M2 microglial polarization. Therefore, we further investigated the mechanism underlying the neuroprotective effects of A2‐Exos on cerebral I/R injury.

Exosomes contain proteins, lipids and miRNAs. miRNAs can target the 3′‐UTR of mRNAs and inhibit the expression of downstream target genes.^34^ Xu et al. demonstrated that astrocyte‐derived exosomes pretreated with oxygen and glucose deprivation (OGD) can be taken up by neurons, thereby reducing OGD‐induced neuronal death and apoptosis by delivering miR‐92b‐3p.^35^ Long et al. demonstrated that miR‐873a‐5p in activated astrocyte exosomes reduces microglia‐mediated neuroinflammation by inhibiting the NF‐κB signalling pathway.^36^ Another study showed that normal astrocyte‐derived exosomes protect neurons by inhibiting MKK4 through the delivery of miR‐200a‐3p.^37^ Thus, miRNAs play crucial roles in the function of astrocyte‐derived exosomes. To further explore the protective mechanism of A2‐Exos after cerebral I/R, we checked the expression level of miR‐628 in the lesion and found a significant increase in miR‐628 expression in A2‐Exos compared to exosomes derived from normal astrocytes or A1‐Exos. Subsequently, we constructed A2‐Exos with overexpression and knockdown of miR‐628 through the transfection of a miR‐628 mimic or miR‐628 inhibitor.

Pyroptosis, a type of cell death in which large amounts of IL‐1β and IL‐18 are released, exacerbates the cascade of inflammatory responses after cerebral I/R injury.^38^ Severe inflammatory responses can aggravate other forms of cell death, including apoptosis.^39^ Moreover, previous studies have shown that NLRP3 is important in inducing cellular apoptosis and promoting the inflammatory response.^40^ Fan et al. showed that during cerebral I/R injury, activated microglia in the central nervous system cause an increase in NLRP3 and pyroptosis‐related proteins. After knocking out the NLRP3 gene, pyroptosis‐related proteins, BBB damage and infarct volume decreased.^41^ Furthermore, an aqueous epimedium extract was shown to ameliorate neuronal pyroptosis by inhibiting NLRP3.^42^ Thus, inhibiting NLRP3 could be an effective treatment for brain I/R injury. In this study, we explored whether miR‐628 decreased the mRNA and protein levels of NLRP3 after cerebral I/R. Dual luciferase reporter assays showed that NLRP3 is a target gene of miR‐628. Compared with A2‐Exos, miR‐628 A2‐Exos inhibited NLRP3 expression at the mRNA and protein levels and cell pyroptosis. In contrast, A2‐Exo inhibitors poorly inhibited NLRP3 and cell pyroptosis. This finding confirms that A2‐Exos alleviated pyroptosis by targeting NLRP3 via miR‐628 delivery.

S1PR3, an S1P receptor, is upregulated after cerebral ischemia.^43^ Compared to that in female patients with IS, the level of the S1PR3 gene was greater in male patients, and the severity of symptoms in male patients indicated that S1PR3 may play a role in the damage caused after cerebral I/R.^44^ Further studies showed that S1PR3 attenuates brain injury after cerebral I/R by regulating OS and is related to BBB damage. After treatment with the S1PR3 inhibitor, OS and damage to the BBB were reduced.^12^, ^45^ These studies suggest that S1PR3 is a potential target for treating cerebral ischemia. In the present study, we found that the mRNA and protein levels of S1PR3 were downregulated following miR‐628 treatment. The dual luciferase reporter assay showed that miR‐628 targeted S1PR3 at the 3′‐UTR. Compared with A2‐Exos, miR‐628 A2‐Exos inhibited S1PR3 and damage to the BBB. Conversely, the inhibitor A2‐Exos poorly inhibited S1PR3 and damaged the BBB, suggesting that A2‐Exos alleviated damage to the BBB by targeting S1PR3 via the delivery of miR‐628.

Astrocytes can be activated by microglia, but the effect of astrocytes on microglia is not yet known.^29^ IRF5 expression in microglia is related to neuroinflammation and poor prognosis.^46^ Previous studies have indicated that IRF5 promotes M1 microglial polarization through nuclear translocation. The M1 and M2 microglial subtypes have proinflammatory and anti‐inflammatory effects, respectively. Inhibiting IRF5 promoted M2 microglial polarization,^16^ indicating that IRF5 is one of the targets of microglial polarization. Our study showed that the mRNA and protein levels of IRF5 were downregulated after miR‐628 treatment. The dual luciferase reporter assay demonstrated that miR‐628 targeted IRF5 at the 3′‐UTR. Compared with A2‐Exos, miR‐628 A2‐Exos inhibited IRF5 and promoted M2 microglial polarization, while the inhibitor A2‐Exos had the opposite effect. These results confirmed that A2‐Exos promoted M2 microglial polarization by targeting IRF5 via miR‐628. Finally, treatment with miR‐628 A2‐Exos significantly reduced cell apoptosis. Our study has several limitations. We grouped two conditionally induced astrocytes based on the available markers C3 and S100A10 and defined them as A1 and A2. This method may not be accurate for determining the properties of astrocytes. Additional investigations are required to explore the properties of reactive astrocytes. This study investigated the effects of only astrocyte‐derived exosomes on the early stages of cerebral I/R injury, and further experiments are needed to evaluate the long‐term impact of these exosomes.

To conclude, we showed that A2‐Exos reduced pyroptosis and BBB damage and promoted M2 microglial polarization through the inhibition of NLRP3, S1PR3 and IRF5 by delivering miR‐628 (Figure S2). Our study explored the mechanism of the intercellular communication of A2‐reactive astrocytes through the exosomal pathway. We demonstrated that miR‐628 could be a potential therapeutic target. Our data suggest that targeting miR‐628 may represent a novel approach for the treatment of IS.

## AUTHOR CONTRIBUTIONS

**Yingju Wang:** Conceptualization (equal); investigation (equal); methodology (equal); writing – original draft (lead). **He Li:** Conceptualization (equal); investigation (equal); methodology (equal). **Hanwen Sun:** Investigation (equal); methodology (equal); validation (equal). **Chen Xu:** Methodology (equal). **Hongxue Sun:** Methodology (equal). **Wan Wei:** Methodology (equal). **Jihe Song:** Formal analysis (equal). **Feihong Jia:** Formal analysis (equal). **Di Zhong:** Conceptualization (equal); funding acquisition (equal); project administration (equal); writing – review and editing (equal). **Guozhong Li:** Conceptualization (equal); funding acquisition (equal); writing – review and editing (equal).

## FUNDING INFORMATION

The present experiment was funded by the Heilongjiang Province Key R&D Program (grant nos. GY2022ZB0104 and GA21C005).

## CONFLICT OF INTEREST STATEMENT

The authors confirm that there are no conflicts of interest.

## Supporting information


**Figure S1.** Experimental design.

**Figure S2.** A2‐Exos reduced pyroptosis and BBB damage and promoted M2 microglial polarization through the inhibition of NLRP3, S1PR3 and IRF5 by delivering miR‐628.

## Data Availability

The data that support the findings of this study are available from the corresponding author upon reasonable request.
