# Supplementary figures and images for: A2 reactive astrocyte‐derived exosomes alleviate cerebral ischemia–reperfusion injury by delivering miR‐628
Source: J Cell Mol Med. 2024 Aug 19;28(16):e70004. doi: 10.1111/jcmm.70004 (PMC11332600; doi:10.1111/jcmm.70004)

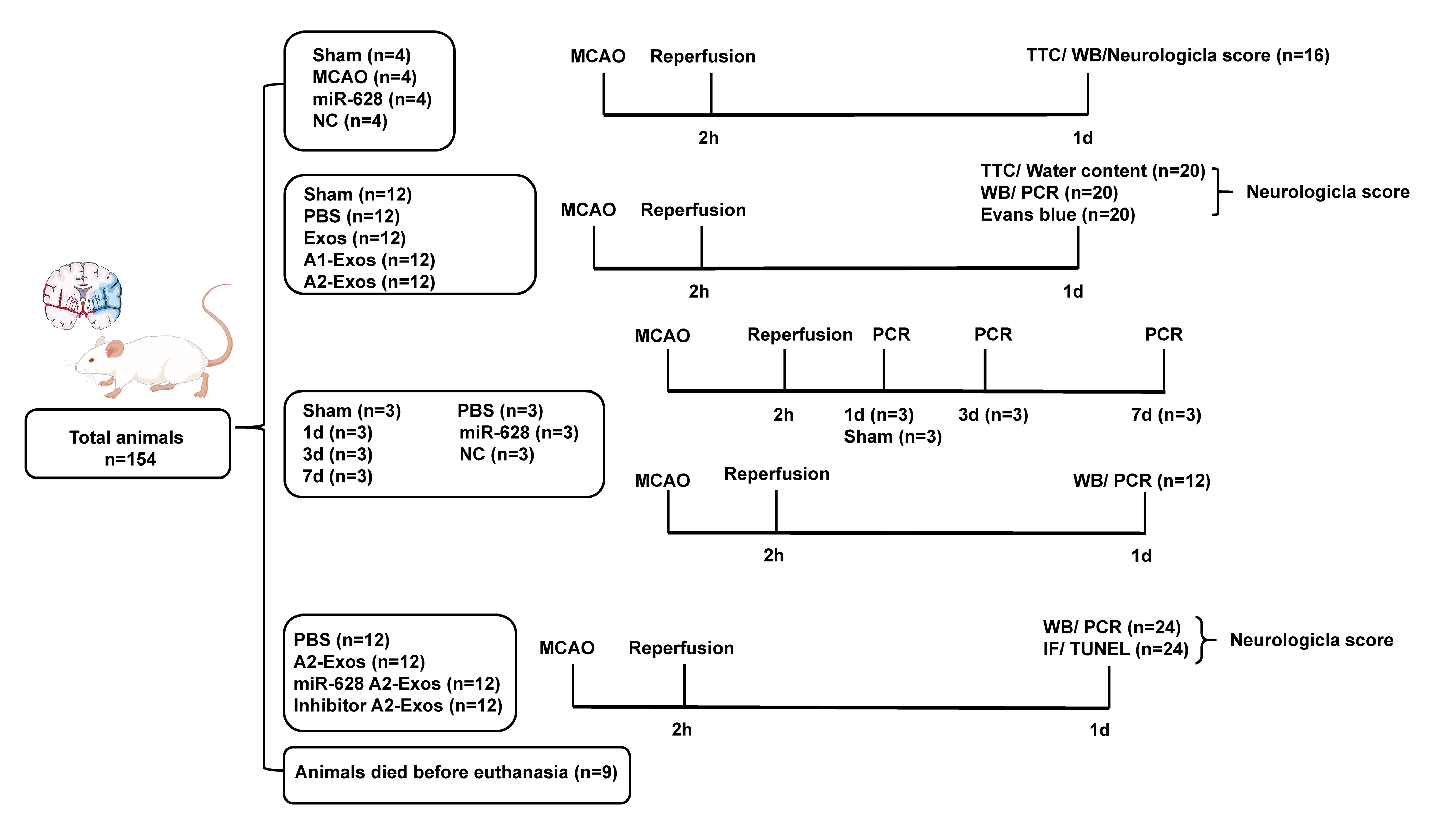

Supplement: Supplementary file 1 — Figure S1. Experimental design. [file JCMM-28-e70004-s002.tif]

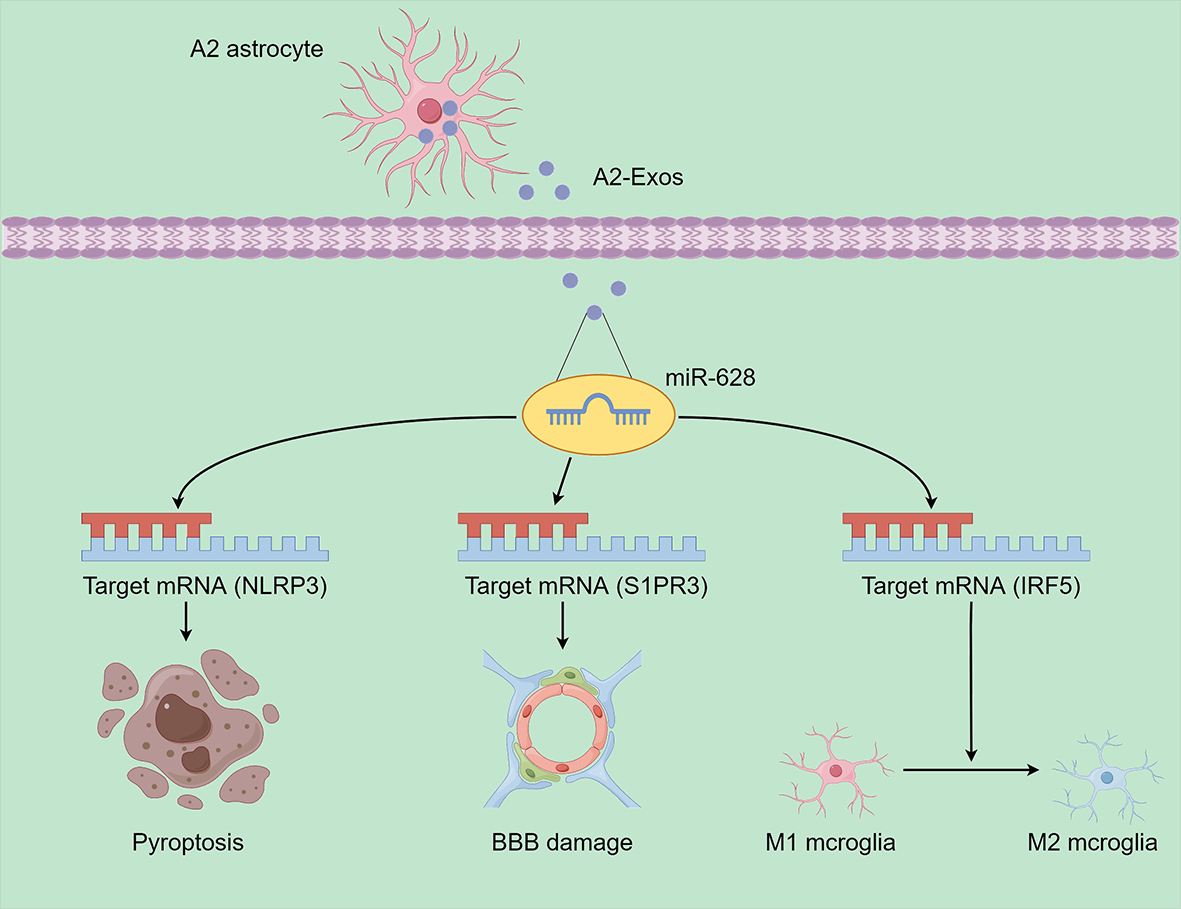

Supplement: Supplementary file 2 — Figure S2. A2‐Exos reduced pyroptosis and BBB damage and promoted M2 microglial polarization through the inhibition of NLRP3, S1PR3 and IRF5 by delivering miR‐628. [file JCMM-28-e70004-s001.tif]
